# Association of *GCK* (rs1799884), *GCKR* (rs780094), and *G6PC2* (rs560887) Gene Polymorphisms with Type 2 Diabetes among Malay Ethnics

**DOI:** 10.1055/s-0042-1760384

**Published:** 2023-01-24

**Authors:** Neda Ansari, Vasudevan Ramachandran, Nur Afiqah Mohamad, Elnaz Salim, Patimah Ismail, Mohamad Hazmi, Liyana Najwa Inchee Mat

**Affiliations:** 1 Department of Biomedical Science, Faculty of Medicine and Health Sciences, Universiti Putra Malaysia, Selangor, Malaysia; 2 Faculty of Health Sciences, University College MAIWP International, Taman Batu Muda, Kuala Lumpur, Malaysia; 3 Centre for Foundation Studies, Lincoln University College, Selangor, DE, Malaysia; 4 Department of Surgery, Faculty of Medicine and Health Sciences, Universiti Putra Malaysia, Serdang, Selangor DE, Malaysia; 5 Department of Medicine, Faculty of Medicine and Health Sciences, Universiti Putra Malaysia, Serdang, Selangor DE, Malaysia

**Keywords:** GCK gene polymorphism, diabetes, biomarkers, Malay ethnics

## Abstract

**Background** Type 2 diabetes mellitus (T2DM) is a complex metabolic disorder, and the underlying causes remain unknown and have not been fully elucidated. Several candidate genes have been associated with T2DM in various populations with conflicting results. The variations found in glucokinase ( *GCK* ), glucokinase regulatory protein ( *GCKR* ), and glucose-6-phosphatase 2 ( *G6PC2* ) genes were not well studied, particularly among Asians.

**Aims** The main objective of this study was to determine the candidate genetic polymorphisms of *GCK* (rs1799884), *GCKR* (rs780094), and *G6PC2* (rs560887) genes in T2DM among Malay ethnics.

**Methods** In this candidate gene association study, a total of 180 T2DM subjects and 180 control subjects were recruited to determine the genotypes using polymerase chain reaction-restriction fragment length polymorphism and *Taqman* probe assay methods. Genotype and allele frequencies in case and control samples were compared using the chi-squared test to determine a significant difference.

**Results** The body mass index, fasting blood glucose, hemoglobin A1c, systolic and diastolic blood pressure, and total cholesterol were significantly different ( *p* < 0.05) between T2DM and control subjects. The genotypic and allelic frequencies of *GCK* (rs1799884), *GCKR* (rs780094), and *G6PC2* (rs560887) gene polymorphisms were significantly different between T2DM and controls ( *p* < 0.05).

**Conclusion** Hence, rs1799884 of *GCK* gene and rs780094 of *GCKR* gene and rs560887 of the *G6PC2* gene are possible genetic biomarkers in T2DM development among Malay ethnics in Malaysia.

## Introduction

Type 2 diabetes mellitus (T2DM) is a complex metabolic disorder recognized with impaired insulin secretion, insulin resistance, and hyperglycemia manifestations caused by the defects in pancreatic b-cells. Although evidence suggests that various environmental and genetic parameters interfere in the development and progression of T2DM, the underlying causes are still unknown and have not been fully elucidated. 1 The most common diabetes, accounting for 90 to 95% of all the diabetes cases, is T2DM reported as a life-lasting metabolic syndrome with a high level of blood sugar as well as the other diabetic complications such as cardiovascular disease, neuropathy, and retinopathy threatening the life. 2 3

According to the latest National Health & Morbidity Survey reports in the year, the prevalence of T2DM in Malaysia was 22.5% in 2015 compared with 20.8% in 2011 and 14.9% in 2006. 4 5 Considering three major ethnicities in Malaysia appears to show a significant variation in the prevalence of T2DM among Malaysians. Among ethnicity, Indians (24.9%) were ranked high compared with the other ethnicities: Malay (16.9%) and Chinese (13.8) in 2011. 6 The main reasons for the rapid development of diabetes among Malaysians include lifestyle alteration due to urbanization and aging, leading to increased obesity and physical inactivity. 7

Although several factors are involved in the development of T2DM, reports suggested that environmental factors such as smoking, alcohol, physical inactivity, and being obese/overweight are the major risk factors characterized in the development of T2DM. 8 Environmental factors affecting T2DM typically are at the roots of genetic susceptibilities. Comprehensive studies have been done to determine the susceptible candidate genes related to T2DM in different populations with contradictory results. Studies have produced various results since genetic varieties exist among inconsistent ethnic populations. However, there is a lack of studies reported about T2DM and susceptibility genes among Malaysian subjects, particularly on Malay ethnics. Apart from environmental factors, genetic factors also play a vital role in the development of T2DM. Genetic polymorphism serves as molecular biomarkers to detect the individual at risk of developing the disease. 9 Studies reported that the genetic variations found in many candidate genes are risk alleles for developing many diseases, including T2DM. In favor of that, many population studies reported the association of genetic polymorphisms and T2DM with significant findings. 10

Glucokinase (GCK) is considered the major enzyme involved in the glucose phosphorylation process as the first rate-limiting step in the glycolysis pathway. In addition, GCK regulates glucose-stimulated insulin secretion, giving attention as a candidate gene for T2DM. 11 Although a variety of candidate genes have been demonstrated to be in association with the development of T2DM in diverse populations, some of the genes such as glucokinase regulatory protein ( *GCKR* ), glucose-6-phosphatase 2 ( *G6PC2* ), and *GCK* are not well studied, particularly among Asians. For instance, the secretion of glucose-stimulated insulin from pancreatic β cells and glucose metabolism has been reported to be regulated by GCK as the main glucose phosphorylation enzyme. 12 A mutation in the promoter region of the *GCK* gene (rs1799884) is shown to cause genetic variation predisposing the risk of T2DM, hyperglycemia, and disruption of β-cell function. 13 14 There is strong evidence indicating the association between rs780094 polymorphism in *GCKR* and metabolic syndrome 15 and decreased risk of susceptibility to T2DM. 16 According to the findings of a large-scale meta-analyses study, the C allele of *GCKR* rs780094 is correlated with an increase in the risk of T2DM. 17 A common *G6PC2* intronic SNP (rs560887) is highly associated with fasting plasma glucose (FPG) and insulin secretion. 14 However, some studies reported that rs560887 single nucleotide polymorphism (SNP) is not associated with T2DM risk 18 and not much reported among Asian populations. To our knowledge, there is a paucity of information on the genetic and allelic frequencies of *GCK* , *GCKR* , and *G6PC2* gene polymorphisms among T2DM, particularly among Malay ethnics. Thus, this study had been conducted to determine the association between the genetic polymorphisms of *GCK* , *GCKR* , and *G6PC2* genes and T2DM subjects among Malay ethnics.

## Method

### Study Design

This case–control study was approved by the National Medical Research Registry, Malaysia (number NMRR-18-547-40575). A total of 360 subjects were recruited based on inclusion and exclusion criteria, including 180 T2DM patients and 180 control subjects without T2DM. All subjects were above 30 years old at the time of recruitment and of Malay ethnicity. T2DM patients were recruited and diagnosed with T2DM by medical practitioners. According to the guidelines from Ministry of Health, Malaysia, T2DM is diagnosed based on FPG of more than or equal to 7.0 mmol/L and hemoglobin A1c (HbA1c) more than or equal to 6.3%. Subjects who were diagnosed with hypertension, type 1 diabetes (T1DM), and gestational diabetes were excluded from the study. Control subjects were free of T2DM, with no family history of T2DM, renal failure, cardiac failure, T1DM, and chronic kidney diseases. A written informed consent and questionnaire were given to all subjects to determine the sociodemographic factors. Biochemical and clinical characteristics such as body mass index (BMI), smoking status, waist-to-hip ratio (WHR), FPG, HbA1c, blood pressure, and lipid profiles were collected from the patient's medical record. A total of 3 mL blood was collected from all subjects for genotyping analysis.

### Genotyping Analysis

Among all the genes studied, rs1799884 of the *GCK* gene was determined using the polymerase chain reaction-restriction fragment length polymorphism (PCR-RFLP) method. The other genetic polymorphisms, *GCKR* (rs780094) and *G6PC2* (rs560887), were determined by *Taqman* probes on a ROTORGENE real-time PCR detection system. Table 1 shows all the primer information and PCR condition of the respective gene polymorphisms of the selected genes. A nontemplate control in which PCR-grade water was used as a template is included in all analysis.

**Table 1:** Information of primers and PCR condition of *GCK* , *GCKR,* and *G6PC* *2* genes

SNPs	Primer	PCR condition
*GCK* (rs1799884)	5′- GGGTTAGGGATGTGAGAT-3′ 5′- GTGGGGCTTAGTGTCCTTC-3′	Initial denaturation- 94°C/5min Denaturation: 94°C/30sec Annealing: 55°C/30sec Extension: 72°C/30sec No. of cycles: 35 PCR product: 224 bp Enzyme: *MwoI* Restricted fragment size: 176 and 224 bp
*GCKR* (rs780094)	rhAmp- F-CATGTTCTCTGAGTCCTTCCA R- AGGCTTGTTGAGAACTCCTGA	Enzyme activation: 95°C/10min Denaturation: 95°C/10sec Annealing: 60°C/30sec Extension: 68°C/20sec Heat inactivation: 99.9°C/15min
*G6PC2* (rs560887)	rhAmp -F GCT TCT TGA AAG GGC AGAGA R − GGA TCA CCT GAG GTC AGG AG	

Abbreviation: PCR, polymerase chain reaction.

### Statistical Analysis

All the continuous variables were represented as mean ± standard deviation, whereas percentages were used for categorical variables. Using descriptive statistics and Student's *t* -test, the biochemical and clinical parameters' mean was determined and compared between groups. Genotypic distribution with Hardy-Weinberg exception was done using the chi-squared test. All statistical analyses with a *p* -value of less than 0.05 was statistically significant. In addition, Confidence Interval was set at 95% in all the tests. For the *Taqman* assay, the genotypes were visualized by dye component fluorescent emission data depicted in the X-Y scatter-plot using SDS software. Statistical Package for the Social Sciences (SPSS) version 21.0 software was used to analyze the data.

## Results

### Study Subjects

Table 2 presents the sociodemographic, anthropometric, and laboratory characteristics of the studied groups. The mean age of the T2DM subjects was 48.62 ± 7.61 years and significantly different from control subjects (40.88 ± 8.97 years, *p* < 0.001), and were in the age range of 40 to 49. The majority of the respondents were male (53%) compared with female (47%) among T2DM subjects. The family history of T2DM was high among cases (71.3%) compared with controls (54.1%). The BMI, FPG, HbA1c, systolic blood pressure, diastolic blood pressure, and cholesterol were highly significant ( *p* < 0.05) between T2DM and control subjects. However, the other risk factors, including smoking, high-density lipoprotein, WHR, and triglycerides, had no significant difference among the subjects ( *p* > 0.05).

**Table 2:** Sociodemographic, anthropometric, and laboratory characteristics of the studied groups

Parameter	Case	Control	*p* -Value
No. of samples	181	181	
Age (years)	48.62 ± 7.61	40.88 ± 8.97	<0.001 a
Gender			
Male	96 (53.0)	85 (47.0)	0.248 b
Female	85(47.0)	96 (53.0)	
Family history			
No	52 (28.7)	83 (45.9)	0.001 b
Yes	129 (71.3)	98 (54.1)	
Smoking			
Current smoking	31 (17.1)	26 (14.4)	0.029 b
Quit smoking	22 (12.2)	9 (5.0)	
Never smoked	128 (70.7)	146 (80.7)	
BMI (kg/m ^2^ )	26.52 ± 4.00	24.71 ± 3.16	<0.001 a
WHR	1.86 ± 9.45	0.96 ± 0.92	0.198 a
FPG (mmol/L)	8.63 ± 1.54	5.36 ± 0.29	<0.001 a
HbA1c (%)	7.87 ± 1.23	4.98 ± 0.78	<0.001 a
SBP (mm Hg)	136.85 ± 16.96	126.75 ± 9.32	<0.001 a
DBP (mm Hg)	83.60 ± 9.12	81.18 ± 5.05	0.002 a
Lipid profile (mmol/L)			
Total cholesterol	4.55 ± 1.07	3.92 ± 1.25	<0.001 a
LDL	2.62 ± 0.77	2.40 ± 0.73	0.007 a
HDL	1.15 ± 0.22	6.91 ± 77.36	0.317 a
TG	2.53 ± 1.06	2.40 ± 1.04	0.260 a

Abbreviations: BMI, body mass index; Chol, cholesterol; DBP, diastolic blood pressure; FPG, fasting plasma glucose; HbA1c, hemoglobin A1c; HDL, high-density lipoprotein; LDL, low-density lipoprotein; SBP, systolic blood pressure; TG, triglyceride; WHR, waist-to-hip ratio.

Data are reported as mean ± standard deviation and number of subjects with percent in parentheses.

a Student's *t-* test.

b Chi-squared test.

### Genotyping Results

The genotypic and allelic distributions among the subjects are shown in Table 3 . The *r* s1799884 of the *GCK* gene, rs780094 of the *GCKR* gene, and rs560887 of the *G6PC2* gene showed significant difference for both genotypes and alleles between T2DM cases and controls ( *p* < 0.05). The post hoc test between T2DM and control subjects also observed significant difference when compared between different genotype groups for all genetic polymorphisms ( *p* < 0.001).

**Table 3:** Genotype and allelic frequencies of *GCK* (rs1799884), *GCKR* (rs780094), and *G6PC2* (rs560887) gene polymorphisms among subjects

SNPs		Genotypes	Control (%)	Case (%)	*p* -Value a
***GCK*** (rs1799884)	Genotypes	AA	21 (11.6)	86 (47.5)	<0.001
		GA	55 (30.4)	64 (35.4)	
		GG	105 (58.0)	31 (17.1)	
	Alleles	A	97 (26.8)	236 (65.2)	<0.001
		G	265 (73.2)	126 (34.8)	
	Post-hoc analysis		χ ^2^		
		AA vs. GA	17.85		<0.001
		AA vs. GG	79.53		<0.001
		GA vs. GG	26.07		<0.001
***GCKR*** (rs780094)	Genotypes	CC	102 (56.4)	56 (30.9)	<0.001
		CT	75 (41.4)	97 (53.6)	
		TT	4 (2.2)	28 (15.5)	
	Alleles	C	279 (77.1)	209 (57.7)	<0.001
		T	83 (22.9)	153(42.3)	
	Post-hoc analysis		χ ^2^		
		CC vs. CT	14.54		<0.001
		CC vs. GG	29.24		<0.001
		CT vs. TT	11.00		<0.001
***G6PC2*** (rs560887)	Genotypes	AA	169 (93.4)	152 (84.0)	0.005
		GA	12 (6.6)	29 (16.0)	
		GG	0 (0)	0 (0)	
	Alleles	A	350 (96.7)	333 (92.0)	0.006
		G	12 (3.3)	29 (8.0)	
	Post-hoc analysis		χ ^2^		
		AA vs. GA	7.95		0.055

Data are reported as the number of subjects with percent in parentheses.

a Chi-squared test.

Gene–age interactions were analyzed by analysis of variance using the General Linear Model procedure. A significant interaction was observed between rs1799884 *GCK* gene polymorphism and age among the T2DM subjects ( *p* = 0.012). However, there were no significant interactions between *GCKR* (rs780094) and *G6PC2* (rs560887) gene polymorphisms with age ( *p* >0.05), as shown in Table 4 .

**Table 4:** Association of *GCK* (rs1799884), *GCKR* (rs780094), and *G6PC2* (rs560887) *GCKGCKRG6PC2* gene polymorphisms with age of the subjects

SNPs	Control				Case			
	Genotype			*p-* Value a	Genotype			*p-* Value a
*GCK* (rs1799884)	AA	GA	GG		AA	GA	GG	
	48.57 ± 7.43	50.42 ± 7.09	47.70 ± 7.81	0.099	39.05 ± 9.05	41.64 ± 8.27	44.39 ± 9.11	0.012*
*GCKR* (rs780094)	CC	CT	TT		CC	CT	TT	
	48.95 ± 8.00	48.74 ± 7.55	48.07 ± 8.86	0.889	40.07 ± 9.50	41.71 ± 8.25	46.00 ± 5.77	0.250
*G6PC2* (rs560887)	AA	GA	GG		AA	GA	GG	
	48.86 ± 7.88	47.86 ± 7.86	–	0.532	40.61 ± 8.58	46.08 ± 13.2	–	0.042

Abbreviation: ANOVA, analysis of variance.

Data are reported as mean ± standard deviation.

a One-way ANOVA test.

* *p* < 0.05, significant.

The three polymorphisms were also compared based on a recessive and dominant genetic model as shown in Table 5 . The *GCK* gene polymorphism observed a significant association with T2DM once the age had been adjusted, based on a logistic regression model. According to the analysis based on the additive model for age, the risk of the A allele of *GCK* gene polymorphism was recorded as high compared with the G allele in association with T2DM (odds ratio = 0.15, confidence interval = 0.09–0.27, *p* = 0.001). The recessive model compared the combined AG + AA genotypes with the homozygous GG, which also recorded a high risk of the A alleles associated with T2DM. The association between the T allele of rs780094 with age and gender was strongly demonstrated in this study ( *p* < 0.01). No significant findings were observed in age and gender among the dominant and recessive models for *G6PC2* gene polymorphism ( *p* > 0.05).

**Table 5:** Odds ratio of T2DM risk factors associated with *GCK* (rs1799884), *GCKR* (rs780094), and *G6PC2* (rs560887) *GCKG6PC2* gene polymorphisms based on genetic models

SNPs	Variable		Model	Test	Control ( *n* )	Case ( *n* )	OR	95% CI	*p* -Value a
*GCK* (rs1799884)	Age		AA + GA vs. GG	DOM	70/99	145/33	0.15	0.09,0.27	<0.001
			AA vs. GA + GG	REC	19/150	89/89	0.17	0.10,0.32	<0.001
			AA + GA vs. GG	DOM	6/6	1/2	–	–	–
			AA vs. GA + GG	REC	2/10	1/2	–	–	–
	Gender	Male	AA + GA vs. GG	DOM	39/57	66/19	0.17	0.08,0.37	<0.001
			AA vs. GA + GG	REC	10/86	41/44	0.20	0.08,0.47	<0.001
		Female	AA + GA vs. GG	DOM	37/48	80/16	0.18	0.09,0.39	<0.001
			AA vs. GA + GG	REC	11/74	49/47	0.18	0.08,0.41	<0.001
*GCKR* (rs780094)	Age		CC + CT vs. TT	DOM	141/27	174/4	0.12	0.04,0.35	<0.001
			CC vs. CT + TT	REC	50/118	100/78	0.33	0.21,0.52	<0.001
			CC + CT vs. TT	DOM	9/1	1/0	0	0	–
			CC vs. CT + TT	REC	5/4	0/1	0	0	–
	Gender	Male	CC + CT vs. TT	DOM	85/0	84/14	0	0	–
			CC vs. CT + TT	REC	25/73	54/31	0.20	0.10,0.37	<0.001
		Female	CC + CT vs. TT	DOM	69/14	92/4	0.21	0.07,0.68	0.009
			CC vs. CT + TT	REC	31/52	48/48	0.60	0.33,1.08	0.090
*G6PC2* (rs560887)	Age		AA + GA vs. GG	DOM	167/1	178/0	–	–	–
			AA vs. GA + GG	REC	141/27	169/9	0.29	0.13,0.64	0.002
			AA + GA vs. GG	DOM	13/0	3/0	–	–	–
			AA vs. GA + GG	REC	11/2	0/3	–	–	–
	Gender	Male	AA + GA vs. GG	DOM	98/0	85/0	–	–	–
			AA vs. GA + GG	REC	85/13	80/5	0.33	0.13,0.84	0.021
		Female	AA + GA vs. GG	DOM	83/0	96/0	–	–	–
			AA vs. GA + GG	REC	67/16	89/7	0.49	0.16,1.49	0.209

Abbreviations: CI, confidence interval; DOM, dominant model; OR, odds ratio; REC, recessive model; T2DM, type 2 diabetes mellitus.

a Logistics regression analysis.

## Discussion

T2DM is a complex multifactorial disease caused by both environmental and genetic factors. Apart from that, the primary risk factors for T2DM are family history, obesity, and physical inactivity. 8 Several research studies have initiated the identification of genetic variants across the entire genome predisposing to T2DM. 19 Through these studies, researchers have identified several T2DM risk loci using several technologies in various populations. The knowledge of the specific causes of common complex diseases at the genetic level remains unclear. Finding the genetic risk factors of T2DM and the underlying molecular mechanisms may progress in preventing the disease, explicate the mechanisms, and pave the way for new pathways relevant for therapeutic and clinical intervention and investigations. This makes it a vital challenge for researchers to identify the susceptibility genes associated with T2DM to elucidate the complex pathogenesis of T2DM. 20

Understanding the genetic polymorphism will able researchers to investigate differences in both clinical manifestations and disease pathways. These include determining the differences among individuals in response to a drug, in the rates at which disease develops, and even in the susceptibility to disease. 9 Accounting for approximately 90% of all the genetic mutations in human DNA, SNP is the most common cause of genetic polymorphism. 21 Analyzing and understanding the pathogenesis of candidate genes provides a more practical approach for identifying and determining the genotype/phenotype and their likely correlation to a disease. 22 Hence, this might lead to discovery of novel treatments and genetic effects on the progression of a disease, particularly for T2DM.

The rs1799884 polymorphism in the *GCK* gene is reported to be associated with the susceptibility to gestational diabetes mellitus (GDM) in a Chinese population 23 and insulin resistance in the Canadian population. 24 The genetic variants of the *GCK* gene were studied mainly in GDM subjects; however, the rs1799884 allele was significantly associated with increased impaired glucose regulation susceptibility. In this study, the genotypic and allelic frequencies of *GCK* (rs1799884) were found to be associated with T2DM among Malay ethnics. The genetic polymorphisms present in *GCKR* gene are also considered genetic risk factors for diabetes. 25 Previous studies have also reported the *GCKR* rs780094 gene polymorphism to be associated with T2DM among the Chinese and Japanese populations. 15 A study reported that C-allele carriers of *GCKR* (rs780094) are 1.22 times more likely to develop T2DM than those with the T allele among the Han-Chinese population. 26 The results of this present study are in accordance with the other population studies, 27 which show a significant difference between the *GCKR* (rs780094) genetic polymorphism and T2DM subjects ( *p* < 0.001).

Several studies have also suggested that the genetic variants found in the *G6PC2* (rs560887) gene influences the fasting glucose levels and decreases insulin secretion during glucose tolerance tests. 28 The observation from the study reports led to the suggestion that *G6PC2* may also regulate insulin secretion and increase the risk of developing T2DM. 29 A strong association was found between rs560887 of the *G6PC2* gene and FPG, 29 similar to findings in the present study and other population studies. 14 30 However, there is contradictory findings in one study that reported no significant association of *G6PC2* (rs560887) polymorphism with T2DM risk. 30 Limitations included in the present study should be taken into consideration. First, the homogeneity of study subjects was drawn only from the Malay ethnicity. Future studies should include the Chinese and Indians, as different ethnicities might have different genetic profiles and T2DM risk. Second, the lack of gene expression data which would provide a better insight on the T2DM risk with candidate genes. However, to our best knowledge, the current study is the first comprehensive findings among T2DM in Malay ethnics. Different genetic profiles could contribute to some of the contrasting findings in the present study with other populations, and these discrepancies might also be related to different study designs and methodologies used. Hence, to further study the effects of various polymorphisms of T2DM candidate genes, the present study needs to be replicated by considering the reported limitations.

## Conclusion

In this study, the genetic variants of *GCK* (rs1799884), *GCKR* (rs780094), and *G6PC2* (rs560887) might be considered a genetic risk factor in T2DM development among Malay ethnicity in Malaysia.
